# Cardiac surgery risk-stratification models

**DOI:** 10.5830/CVJA-2011-047

**Published:** 2012-04

**Authors:** Carla Prins, I De Villiers Jonker, Francis E Smit, Lizelle Botes

**Affiliations:** Department of Cardiothoracic Surgery, University of the Free State, Bloemfontein, South Africa; Department of Cardiothoracic Surgery, University of the Free State, Bloemfontein, South Africa; Department of Cardiothoracic Surgery, University of the Free State, Bloemfontein, South Africa; School of Health Technology, Central University of Technology, Bloemfontein, South Africa

**Keywords:** risk-stratification models, open-heart surgery, intra- and postoperative variables, surgical outcomes

## Abstract

**Abstract:**

Risk models are widely used to predict outcomes after cardiac surgery. Not only is risk modelling applied in the assessment of the relative impact of specific risk factors on surgical outcomes, but also in patient counselling, the selection of treatment options, comparison of postoperative results, and quality-improvement programmes. At least 19 risk-stratification models exist for open-heart surgery. The focus of risk models was originally on pre-operative prediction of mortality. However, major morbidity is in general more common than mortality and the ability to predict only operative mortality is not an adequate method of determining surgical outcome. Multiple intra- and postoperative variables have been excluded in the majority of models and the possible effect of their future inclusion remains to be seen. The unique patient population of sub-Saharan Africa requires a unique risk model that reflects the patient population and levels of care.

## Abstract

Risk models are widely applied in the assessment of the relative impact of specific risk factors on surgical outcomes. These models enable surgeons to select the ideal treatment option for a specific patient and to counsel patients accordingly. They allow for comparison of postoperative results and assist in assessment of quality-improvement programmes.^1^,^2^

One of the original aims for the development of cardiac risk models was risk adjustment, allowing fair comparison of treatment outcomes among different institutions or surgeons.^2^ Risk models were then also applied in clinical decision making, advising individual patients of their peri-operative risk, quality-improvement programmes comparing year-to-year outcomes, as well as allocation of healthcare resources through the prediction of length of stay and postoperative complication rates.^1^,^3^

The first widely used risk model, the Parsonnet score, was based on a retrospective analysis of data collected during the 1980s.^1^,^4^ Risk modelling since then has been significantly influenced by advances made in diagnostic and interventional technology. The advances in interventional cardiology are believed to have adversely changed the risk profile of patients presenting for cardiac surgery. A greater number of elderly patients, those with associated illnesses, and patients presenting for re-operation are now seen.^3^,^5^

At least 19 risk-stratification models exist for open-heart surgery.^4^ These models are summarised in Table 1.

**Table 1:** A Summary Of Cardiac Surgery Risk-Stratification Models^4,31^ (With Permission)

*Model*	*Region*	*Year of data collection*	*Year of publication*	*Number of patients (centres)*	*Risk variables*
Amphiascore	Netherlands	1997-2001	2003	7 282 (1)	8
Cabdeal	Finland	1990-1991	1996	386 (1)	7
Cleveland Clinic	USA	1986-1988	1992	5 051 (1)	13
EuroSCORE (additive)	Europe	1995	1999	13 302 (128)	17
EuroSCORE (logistic)	Europe	1995	1999	13 302 (128)	17
French score	France	1993	1995	7 181 (42)	13
Magovern	USA	1991-1992	1996	1 567 (1)	18
NYS	USA	1998	2001	18 814 (33)	14
NNE	USA	1996-1998	1999	7 290 (N/A)	8
Ontario	Canada	1991-1993	1995	6 213 (9)	6
Parsonnet	USA	1982-1987	1989	3 500 (1)	16
Parsonnet (modified)	France	1992-1993	1997	6 649 (42)	41
Pons	Spain	1994	1997	1 309 (7)	11
STS risk calculator*	USA	2002–2006	2007		
isolated CABG				774 881 (819)	49
valve procedures				109 759	50
CABG and valve				101 661	50
Toronto	Canada	1993-1996	1999	7 491 (2)	9
Toronto (modified)	Canada	1996-1997	2000	1 904 (1)	9
Tremblay	Canada	1989-1990	1993	2 029 (1)	8
Tuman	USA	N/A	1992	3 156 (1)	10
UK national score	UK	1995-1996	1998	1 774 (2)	19
Veterans Affairs	USA	1987-1990	1993	12 712 (43)	10

USA = United States of America, EuroSCORE = European System for Cardiac Operative Risk Evaluation, NYS = New York State, NNE = Northern New England, STS = Society of Thoracic Surgeons, CABG = coronary artery bypass graft surgery, UK = United Kingdom. *The STS risk calculator consists of seven risk-prediction models in three main categories, namely isolated CABG, valve procedures, and combined CABG and valve procedures. Data represented for the STS risk calculator reflect the number of patients and risk variables captured in the database used for the latest models developed (version 2.61).

The focus of risk models was originally on pre-operative prediction of mortality. However, major morbidity is in general more common than mortality, and the ability to predict only operative mortality is not an adequate method of determining surgical outcome.^6^ Risk modelling has therefore now in some instances, for example the STS score, been expanded to also allow for the calculation of postoperative morbidity.^1^

The assessment of variables that may affect patient outcome, which are not necessarily related to pre-operative patient characteristics, are also often not taken into account. These variables include factors related to the skill and experience of the surgical and postoperative care teams, which in turn influence various aspects of the intra-operative and immediate postoperative period.^1^ Knowledge of adverse intra-operative events has been shown to enhance pre-operative risk prediction, and it is reasonable and necessary to include these variables in risk models.^7^

Cardiac risk models are generally comparable with regard to the pre-operative risk factors included. The most widely used models (e.g. EuroSCORE) were usually designed for various cardiac surgical procedures and cannot necessarily account for co-morbid diseases and aspects of the underlying pathophysiology/disease progression not included in the calculation of risk.^1^ However, over-complication of models has also received a lot of criticism from strong supporters of the concept that ‘simple models will sometimes outperform more complex models...’.^8^ Nevertheless, when the problem is complex, deliberate limitation of the complexity of a model may be unproductive.^8^

The objective of this article is to provide a review of the most common currently used risk-stratification models in cardiac surgery, with critique in general that relates to practice in sub-Saharan Africa.

## Currently used models

There are a number of risk-stratification models in cardiac surgery. Three of the most widely used models, applicable to multiple cardiac procedures, include the European System for Cardiac Operative Risk Evaluation (EuroSCORE), the Society of Thoracic Surgeons (STS) algorithms, and the Parsonnet score.1 These will be briefly discussed.

## The European System for Cardiac Operative Risk Evaluation

Combining the most important pre-operative risk factors, the EuroSCORE method has been shown to be a valuable measure for prediction of immediate death after adult cardiac surgery.^9^ It has been studied widely and is believed by many to be the gold standard.^1^

The clinical aim of the logistic EuroSCORE was to construct a scoring system predicting early mortality in cardiac surgical patients in Europe on the basis of objective risk factors.^3^ It was developed from a large European database and eventually included 13 302 patients.^3^ Prospective data collection took place in eight European countries between September and December of 1995.^10^

The EuroSCORE provides two methods for calculating predicted outcome: the additive model and the logistic model.^3^ Validation of the EuroSCORE took place all over the world in a variety of population settings.^1^,^11^,^12^ The logistic EuroSCORE uses logistic regression and the risk has to be calculated in a very complex way. The simpler additive model was derived from the full logistic model by approximating the odds ratios (OR) or modified coefficients from the logistic equation with integers, which can then be added together at the bedside to provide a useful estimate of risk in an individual patient.^13^,^14^

Although well established and validated in patient populations, the additive EuroSCORE sometimes underestimates the risk when certain combinations of risk factors co-exist.^14^ The logistic EuroSCORE on the other hand, has been reported by various centres to over-predict risk despite gradual worsening of the risk profiles of patients and the improvement in cardiac surgical outcomes observed.^15^ Although the additive model is easier to use, the logistic EuroSCORE has been reported to have a better risk-predictor ability, especially in high-risk patient groups.^16^,^17^ The logistic EuroSCORE lacks the prediction of possible morbidity and does not include any intra-operative variables.

Evidence that the EuroSCORE might be out of date led to the collection of new data to enable re-evaluation. Data collection started early in 2010. It was estimated that if enough centres participated, data collection would only take three weeks, but the longest period asked for would be three months.^18^

## The Society of Thoracic Surgeons Algorithms

The Society of Thoracic Surgeons National Cardiac Database (STS NCD) was created in 1989 and it has become the largest clinical database of its kind. The primary aim for the development of the STS model was the support of national quality-improvement programmes. Now it is also used for research focusing on improvement of patient care and outcome.^19^

The STS NCD is unparalleled in terms of its size and comprehensiveness: data were collected prospectively from more than 950 participating centres in the United States.^3^,^20^ The STS NCD now also includes more than 3.6 million surgical procedures.^20^

STS risk models for various cardiac procedures have been developed since 1999 and have undergone periodic revisions.^1^,^20^ A wide variety of endpoints are included in some of the models calculating risk for isolated coronary artery bypass grafting, valve surgery or combined surgeries.^1^ Twenty-seven new STS adult cardiac surgery models for 2008 have been developed and validated.^21^

The predictive performance of the STS algorithms is in general comparable with other systems and remains the most widely used model in the United States.^1^,^3^ The STS NCD also does not predict possible morbidity and does not include relation to any intra-operative variables.

## Parsonnet score

The Parsonnet score was first described in 1989 by Victor Parsonnet. The aim was to construct a straightforward uniform reporting system for levels of operative mortality risk in all cardiac surgical procedures, which included data that are readily available. It includes objective risk factors in order to leave little room for bias.^3^

Development took place in the United States and included data from 3 500 patients collected between 1982 and 1987. Retrospectively, analyses included uni- and multivariate logistic regression models. The model was prospectively tested in an additional 1 332 procedures at a single site. A second, additive model was also developed. This method was tested at two other centres and the outcomes were comparable to those of the hospitals.^3^

The Parsonnet score received widespread acceptance, but the predictive accuracy has been diminished as a result of advances in treatment.^1^ The original score was later modified in 1994 to include 30 new risk factors, according to the SUMMIT system, and is known as the ‘modified Parsonnet score’.^22^ Again, no morbidity or relation to intra-operative events are being predicted.

## Major critique of current models

In recent years, several models have predicted a rising probability of operative mortality while the observed mortality has decreased.^23^ This is due to an increasing prevalence of high-risk patients, believed to be attributed to significant advances made in diagnostic and interventional cardiology.^3^,^5^ Risk models from earlier periods (or retrospectively collected data) can as a result not be used when the goal of the outcome analysis includes determination of the trend of mortality over time. Retrospective data do not only fail to take into calculation the advances in treatment, but also the evolution of the case mix. Therefore, the gold standard for data collection should be speciality-specific, prospectively maintained clinical databases that ought to contain a core set of variables that have been demonstrated to be associated with outcome.^24^

It is furthermore believed that risk models usually predict outcome more accurately in the setting where it was originally developed.^5^ Socio-economic conditions, living standards, healthcare funding, and geographic and ethnic origins affect the applicability of risk models in different regions.^3^ To date no sub-Saharan African country has developed a risk-stratification model applicable to the unique pathology of their native population.

Risk models have diverse clinical aims. The choice of inclusion/exclusion of risk factors as well as the number of risk factors included in the model is influenced by the clinical aim.^25^ Variables that may affect patient outcome but which are not necessarily related to pre-operative patient characteristics are often not taken into account. These include variables related to adverse intra-operative events as well as co-morbid diseases and aspects of the disease progression not included in the calculation of risk.^1^ There is no general agreement about the inclusion and exclusion of these factors.^8^

Risk factors associated with outcomes generally are likely to reflect concurrent, disease-specific variables whereas factors associated with increased resource utilisation reflect serious co-morbid disease.^26^ It has been suggested that the strength of scores should be that some kind of grouping is provided for patient cohorts.^27^

Models are sometimes criticised for multicollinearity. Intercorrelations between independent variables included in risk models are known as multicollinearity (e.g. obesity and diabetes mellitus). Including large numbers of independent variables increases the risk of multicollinearity and the consequent inclusion of redundant information in the model.^8^

Excessively complex models with too many variables will appear to have an extremely good fit in the training set, but generalise poorly to test samples and have limited predictive abilities. This is known as overfitting.^18^ It is recommended that instead of including all statistically significant variables, one should confine the model to the most powerful predictors or combinations of variables that are the most powerful predictors.^8^

Different operators will provide different interpretations to categorical risk factors, such as chronic obstructive pulmonary disease and unstable angina. Even with clearly stated definitions, a degree of personal interpretation takes place, resulting in different final risk scores.^8^ Wherever practical, continuous data should be used and there should be strict standardisation of definitions for the risk factors and the outcomes measured.^18^

Some models have been criticised for not being able to predict individual risk. Currently utilised models are derived from the studies of very large populations and although very effective at predicting population outcomes, are not necessarily suited for the prediction of risk of an individual patient.^1^,^26^ As previously stated, it is generally accepted that the number of independent variables that can be included in a multivariate logistic regression depends on the number of events: there should be a variable-to-event ratio of 1:10 .^8^,^25^ For that reason, to contemplate a 15 risk-factor model with a mortality rate of 3%, at least 5 000 cases are required to achieve adequate sample size.^25^ This also means that even in a unit performing 500 surgeries per annum, it would take at least 10 years to meet the required sample size.

Most of the scores are unsuitable for individual risk prediction despite the sample size. This is due to a simple methodological reason: the application of logistic regression models mathematically describes a multiphasic, more complex behaviour of a survival curve that cannot achieve enough statistical power to achieve enough statistical accuracy for individual predictions.^27^

Lastly, the focus of cardiac risk models was originally on pre-operative prediction of mortality, but complications and potentially preventable morbidity are also important outcomes.^1^,^3^ Ideally, a range of outcomes should be reported: mortality, morbidity, changes in functional status and quality of life, cost of care as well as patient-reported perceptions of the non-technical aspects of care.^3^

## Discussion

Open-heart surgery is one of the most expensive surgical procedures in a hospital. The cost of surgery can vary enormously between patients with an uncomplicated recovery and those who suffer from postoperative complications.^28^

Risk stratification is not only essential for improvement of surgical outcomes, but also allows quality analysis and meaningful comparison of outcomes. Kolh (2006) stated that it should be an integral part of cardiac surgical practice, and quoted ‘... being as essential to the surgeon as the knowledge of anatomy and techniques’.

Clinical research and treatment strategies of cardiovascular disease as well as risk-prediction models have largely been developed in North America and Europe. However, the applicability of results derived from these investigations is unknown.^29^ Popular risk models have been studied extensively around the world. Of these models, the European System for Cardiac Operative Risk Evaluation (EuroSCORE) has been validated in different population settings and remains for many, the gold standard.^1^,^12^

Even though the mortality outcome predicted with the EuroSCORE seems applicable in South African practice, different stages of epidemiological transition are often at work in South Africa and changing patterns in the development of cardiovascular disease are observed in the various ethnic populations.^11^,^30^ Predictions of postoperative recovery in the South African setting are therefore less well established. Given the economic impact of interventional therapy and complications related to intervention, it is incumbent on clinicians in South Africa to ensure the optimal application of interventional therapy and resource allocation.

As a result, cardiac surgeons in South Africa face three options with regard to risk stratification: to simply use external risk scores, knowing that the identified risks and attributed weights might not correctly reflect their patient population; to adjust the weight of the risk factors on the basis of their own data; or to derive a new internal model from their own data and recalibrate it periodically.^8^ Despite continuous research, no perfect risk-prediction model exists and the shortcomings of the different models and criticism of the modelling processes have been comprehensively discussed.^1^,^3^,^25^

Variables that may affect patient outcome but which are not necessarily related to pre-operative patient characteristics, are often not taken into account. These include the skill and experience of the surgical and postoperative care team, which influences various aspects of the intra-operative and immediate postoperative period.^1^ For that reason, current risk-stratification models can only score the risk of care and not the quality of suitable care.

## Conclusion

It is our hypothesis that the development of an integrated model that includes hitherto unutilised intra-operative risk factors as well as other known peri-operative risk factors predictive of outcome should enable more accurate risk stratification and consequently improved quality management of surgical treatment of patients with cardiovascular disease in the South African setting. Such a model would allow for improved clinical decision making, assessment of surgical performance and quality of care. Increasing efficiency through prediction of postoperative complications would ultimately facilitate decisions to operate, allocate resources and estimate costs.^28^
